# Potential of serum sulfatide levels as a marker for classification and disease activity in lupus nephritis

**DOI:** 10.3389/fimmu.2025.1571147

**Published:** 2025-06-16

**Authors:** Kosuke Yamaka, Daiki Aomura, Makoto Harada, Takero Nakajima, Takayuki Nimura, Koji Hashimoto, Naoki Tanaka, Yuji Kamijo

**Affiliations:** ^1^ Department of Nephrology, Shinshu University School of Medicine, Matsumoto, Japan; ^2^ Center for Medical Education and Clinical Training, Shinshu University School of Medicine, Matsumoto, Japan; ^3^ Department of Global Medical Research Promotion, Shinshu University School of Medicine, Matsumoto, Japan; ^4^ International Relations Office, Shinshu University School of Medicine, Matsumoto, Japan; ^5^ Research Center for Social Systems, Shinshu University School of Medicine, Matsumoto, Japan

**Keywords:** lupus nephritis, sulfatides, sphingolipids, systemic lupus erythematosus, vasculitis

## Abstract

**Introduction:**

Lupus nephritis (LN) is a severe complication of systemic lupus erythematosus, often leading to end-stage kidney disease. Serum sulfatide levels are linked to severe kidney vasculitis. This study aimed to assess serum sulfatide levels as a marker for classifying and evaluating disease activity in LN.

**Methods:**

We conducted a retrospective study of patients admitted to our hospital between 2003 and 2022. Serum sulfatide levels were compared between LN patients and controls as well as across LN classes based on the International Society of Nephrology/Renal Pathology Society classification. We also analyzed the association between sulfatide levels and active lesions, the Activity Index, and its components.

**Results:**

Serum sulfatide levels were significantly lower in LN patients than in controls (6.90 ± 2.22 vs. 8.34 ± 1.68, *P* = 0.007). Levels across LN classes were as follows: 9.41 nmol/mL in Class I, 8.21 ± 1.68 nmol/mL in Class II, 7.33 ± 2.25 nmol/mL in Class III, 6.14 ± 1.63 nmol/mL in Class IV, and 7.89 ± 2.12 nmol/mL in Class V, with Class IV having the lowest levels. Serum sulfatides were significantly lower in patients with active lesions (6.38 ± 1.81 vs. 8.23 ± 2.55, *P* = 0.006) and negatively correlated with the Activity Index (r = −0.51, *P* < 0.001) and pathological components such as endocapillary hypercellularity, neutrophils/karyorrhexis, and interstitial inflammation (*P* < 0.001).

**Conclusions:**

Serum sulfatide levels were significantly lower in LN patients than in controls and strongly correlated with active lesions and the Activity Index. These findings suggest sulfatide levels as a useful marker for assessing LN disease activity.

## 1 Introduction

Lupus nephritis (LN) occurs in approximately 30%–50% of systemic lupus erythematosus (SLE) patients and remains one of the most severe complications (1). Despite advancements in immunosuppressive therapy, 10%–20% of patients progress to end-stage kidney disease (ESKD) (2, 3). LN is classified into six classes (I–VI) based on pathological findings, with each class having distinct pathology and therapeutic approaches (4, 5). Additionally, evaluating both active and chronic histopathological features is crucial for assessing disease activity, renal prognosis, and reversibility, making kidney biopsy essential (4, 6). However, performing a timely kidney biopsy can be difficult owing to factors, such as poor systemic health, pancytopenia, or coagulation issues. Moreover, LN often relapses and sometimes switches classes (6, 7), requiring repeat biopsies to adjust treatment strategies. Therefore, noninvasive methods for assessing LN are sought to reduce patient burden, but few novel biomarkers have been identified as alternatives to kidney biopsy.

3-O-sulfogalactosylceramides (sulfatides) are glycosphingolipids composed of ceramide, galactose, and sulfate (8–10). Serum sulfatides (SS) play a role in regulating inflammation and thrombogenesis in blood vessels (11). In our previous studies, SS levels were significantly lower in patients with various types of vasculitis, including IgA vasculitis (IgAV), anti-neutrophil cytoplasmic antibody (ANCA)-associated vasculitis (AAV), and anti-glomerular basement membrane disease (GBM), than in healthy controls (12–14), with lower SS levels linked to active pathological findings on kidney biopsy (12, 14). Given that LN is partly considered a form of kidney vasculitis primarily affecting the kidney microvasculature, SS may also be associated with the histopathological classification and activity of LN.

To explore this, we measured SS levels in LN patients and examined their correlation with pathological classification and disease activity based on kidney biopsy findings.

## 2 Materials and methods

### 2.1 Study design

This retrospective, single-center, observational study was conducted at Shinshu University Hospital, Japan. We enrolled patients diagnosed with SLE who underwent kidney biopsy at our hospital between January 1, 2003, and December 31, 2022. To measure SS, we used residual serum from routine blood tests taken at the time of kidney biopsy, which was frozen at −80°C. The exclusion criteria of this study were as follows: 1) aged < 18 years at admission, 2) residual serum at kidney biopsy not available, and 3) diagnosed with a condition other than LN on kidney biopsy. For comparison with healthy controls, we used serum samples from living kidney donors who underwent transplantation at our hospital between 2008 and 2022. All donors underwent medical screening within 1 year before surgery, and those who passed the tests and completed the procedure by December 31, 2022, were included in the study. SS levels were measured in frozen serum obtained during pre-transplant screening. All clinical data, including serological parameters, urinalysis findings, and background information on comorbidities for the donor group, are identical to those reported in our previous publication (12, 14). We measured SS levels and analyzed their association with the International Society of Nephrology/Renal Pathology Society (ISN/RPS) classification and LN disease activity.

### 2.2 Collection of patient data

Patient data, including age, sex, body mass index (BMI), ethnicity/race, coexistence of hypertension and diabetes mellitus, vital signs, physical findings, and laboratory data at admission, were collected from hospital medical records. Hypertension was defined as a prescription for antihypertensive medication and/or a history of hypertension as described in the medical records. Diabetes mellitus was defined as an elevated hemoglobin A1c level (>6.5%), insulin or hypoglycemic agent prescription, and/or a history of diabetes mellitus listed in the medical records. All individuals with diabetes in the donor group were enrolled only after confirming the absence of clinical or histological evidence of kidney involvement. The Systemic Lupus Erythematosus Disease Activity Index (SLEDAI), a scoring system for SLE activity, was assessed using data from medical records (15). Serositis refers to pericarditis, pleuritis, and peritonitis, while pulmonary involvement was defined as interstitial lung disease, alveolar hemorrhage, or pulmonary hypertension due to SLE. The estimated glomerular filtration rate (eGFR) was calculated using a previously reported formula (16). For each patient, information on death; kidney outcomes, including ESKD and a continuous decline in eGFR of > 30% compared with baseline levels at the 1-year and 3-year follow-up (ΔeGFR < -30%); SLE flare; and thrombotic complications was collected until December 31, 2023. ESKD was defined as persistence of eGFR < 15 mL/min/1.73m^2^ or requirement of dialysis or kidney transplantation. A SLE flare was defined as worsening of SLE activity requiring intensified treatment in a hospital setting. Thrombotic complications included deep vein thrombosis, kidney vein thrombosis, pulmonary embolism, stroke, and myocardial infarction.

### 2.3 Definition and classification of pathological findings on kidney biopsy

Kidney pathology findings for LN patients were extracted from pathology reports prepared by the hospital’s kidney pathology specialists. Pathological classification and activity assessment were performed according to the ISN/RPS classification (17). Patients with overlapping Class V in Classes III or IV were categorized as belonging to Class III or Class IV, respectively. Active lesions and chronic lesions were evaluated in both Class III and IV. Active lesions refer to endocapillary hypercellularity within capillary loops, fibrinoid necrosis, karyorrhexis, cellular/fibrocellular crescents, hyaline thrombi, and wire-loop lesions. Chronic lesions include glomerular sclerosis, fibrous crescents, interstitial fibrosis, and tubular atrophy. Patients with only active lesions were classified as group A, those with only chronic lesions as group C, and those with both lesion types as group A/C. The Activity Index (A-index) and Chronicity Index (C-index) were used to semi-quantitatively evaluate A and C lesions, based on the modified NIH indices from the 2018 proposed revision (4). The components of the A-index include endocapillary hypercellularity, leukocyte infiltration, fibrinoid necrosis, karyorrhexis, cellular/fibrocellular crescents, hyaline thrombi, and interstitial inflammation. The components of the C-index include glomerular sclerosis, fibrous crescents, tubular atrophy, and interstitial fibrosis. Each component comprising these scores was evaluated as follows: 0 = not present, 1+ = present in 1%–25% of glomeruli or tubulointerstitium, 2+ = present in 26%–50% of glomeruli or tubulointerstitium, or 3+ = present in > 50% of glomeruli or tubulointerstitium. For fibrinoid necrosis and cellular/fibrocellular crescents, the scores doubled as revised. The scores for each component are summed to calculate the index. Data for all pathological findings were obtained from the pathology reports mentioned above.

### 2.4 Measurement of the SS level

SS levels were measured using matrix-assisted laser desorption ionization–time of flight mass spectrometry, as described in our previous study (18), with minor modifications (12–14). Briefly, 50 µL of serum from patients was mixed with 18 volumes of n-hexane/isopropanol solution (3:2, v/v) for total lipid extraction. Pooled normal human serum (#12181201, lot#BJ10633A, Cosmobio, Tokyo, Japan) was used as the standard, and total lipids were extracted in the same manner. The sulfatide concentration in the pooled human serum was determined prior to the study. Lipid extracts from samples and standard serum were treated with methanolic sodium hydroxide, heated to convert sulfatides to lysosulfatides (LS), and purified using Monotip C18 cartridges (GL Sciences, Tokyo, Japan). In this process, lysosulfatides refer to the fatty acid–free form of sulfatides, in which the fatty acid chains are removed, leaving only the sphingoid base backbone. This eliminates the variability introduced by differences in fatty acid length or composition, allowing for more consistent comparison across samples. Moreover, lysosulfatides are highly ionizable and structurally simpler, making them particularly suitable for reliable quantification using mass spectrometry. Equal amounts of N-acetylated LS-sphinganine (LS-d18:0-NAc) calibrator were added to each sample. After drying, the LS samples were dissolved in a 9-aminoacridine matrix solution (5 mg/mL in 80% methanol) and spotted onto a matrix-assisted laser desorption ionization–time of flight mass spectrometry plate. The analysis was performed using a TOF/TOF 5800 system (AB Sciex, Framingham, MA, USA) in the negative ion reflector mode with 2-point external calibration. Seven LS species were detected in normal human serum, with LS-sphingadienine (d18:2), d18:1, and phytosphingosine (t18:0) being the major species, comprising over 80% of the total detected (19). In the present study as well, the remaining four species were scarcely detectable deemed negligible in terms of intergroup differences; therefore, we focused on the three major LS species, and their concentrations were calculated using the standard serum data, consistent with our previous approach (12–14). The sum of LS-d18:2, d18:1, and t18:0 concentrations was defined as the serum sulfatide concentration. Each sample was prepared in duplicate or triplicate, with at least two spots analyzed per replicate. In this study, we compared the total SS levels and proportions of major species (d18:2, d18:1, and t18:0) across study groups.

### 2.5 Statistical analysis

Continuous variables were compared between two groups using Student’s t-test or the Mann–Whitney U test, as appropriate. Categorical variables were compared using Fisher’s exact test. To compare continuous variables across more than two groups, one-way analysis of variance (ANOVA) or the Kruskal–Wallis test was used, depending on data distribution. In comparing SS levels across Classes I–V and the donor group, as well as groups A, A/C, and C, an initial one-way ANOVA was performed to assess overall group differences. Subsequently, pairwise comparisons were conducted using univariate and multivariate logistic regression analyses, adjusting for age and sex. For Class V cases, mixed cases (III + V and IV + V) were classified as Class III and Class IV, respectively, in the analysis. The SS species in each disease group were compared with those of healthy controls. SS levels were correlated with SLE activity parameters using Pearson or Spearman correlation analysis, as appropriate. The relationship between pathologically active lesions and SS levels was evaluated using logistic regression analysis, adjusting for age, sex, eGFR, and albumin levels. The detection ability of SS levels for active lesions was assessed using receiver operating characteristic (ROC) curve analysis to calculate the area under the curve (AUC). The AUC for SS levels was compared with those of other potential predictors, including double-stranded DNA antibodies (ds-DNA), complement 3 (C3), complement 4 (C4), eGFR, urinary total protein-to-creatinine ratio (U-TP/Cr), and SLEDAI, using DeLong’s test.

The detection ability of combined formulas, created using SS levels and each predictor, was also evaluated. Each combined formula was constructed using a logistic regression model to maximize the AUC value. The AUC of combined formulas was compared with that of each predictor alone. The cut-off value, sensitivity, and specificity were determined using the Youden index. The exact R formulas used to construct these models are summarized in **Supplementary Table 1**. The correlation between SS levels and the A-index/C-index was analyzed using Pearson or Spearman correlation analysis. The mean SS level for each score component of the A-index was calculated, and trends were assessed using the Jonckheere–Terpstra trend test.

Univariate logistic regression analyses were used to evaluate the relationship between SS levels and clinical outcomes 1 and 3 years after admission. To account for variability in SS measurements, adjustments were made for the exam date using a linear regression model, with SS level as the dependent variable and exam date as the independent variable. Missing values for C4 and dsDNA levels were substituted with plausible values using the univariate imputation method from the mice 3.15 package in R. The proportion of missing values was less than 5%. Statistical significance was set at *P* < 0.05, and all analyses were performed using R software (version 4.2.2).

### 2.6 Ethical approval

This study was approved by the Shinshu University School of Medicine Ethics Committee (approval number: 5768) in accordance with the Declaration of Helsinki. The requirement for written informed consent was waived owing to the retrospective nature of the study.

## 3 Results

### 3.1 Patient characteristics

**Figure 1:**
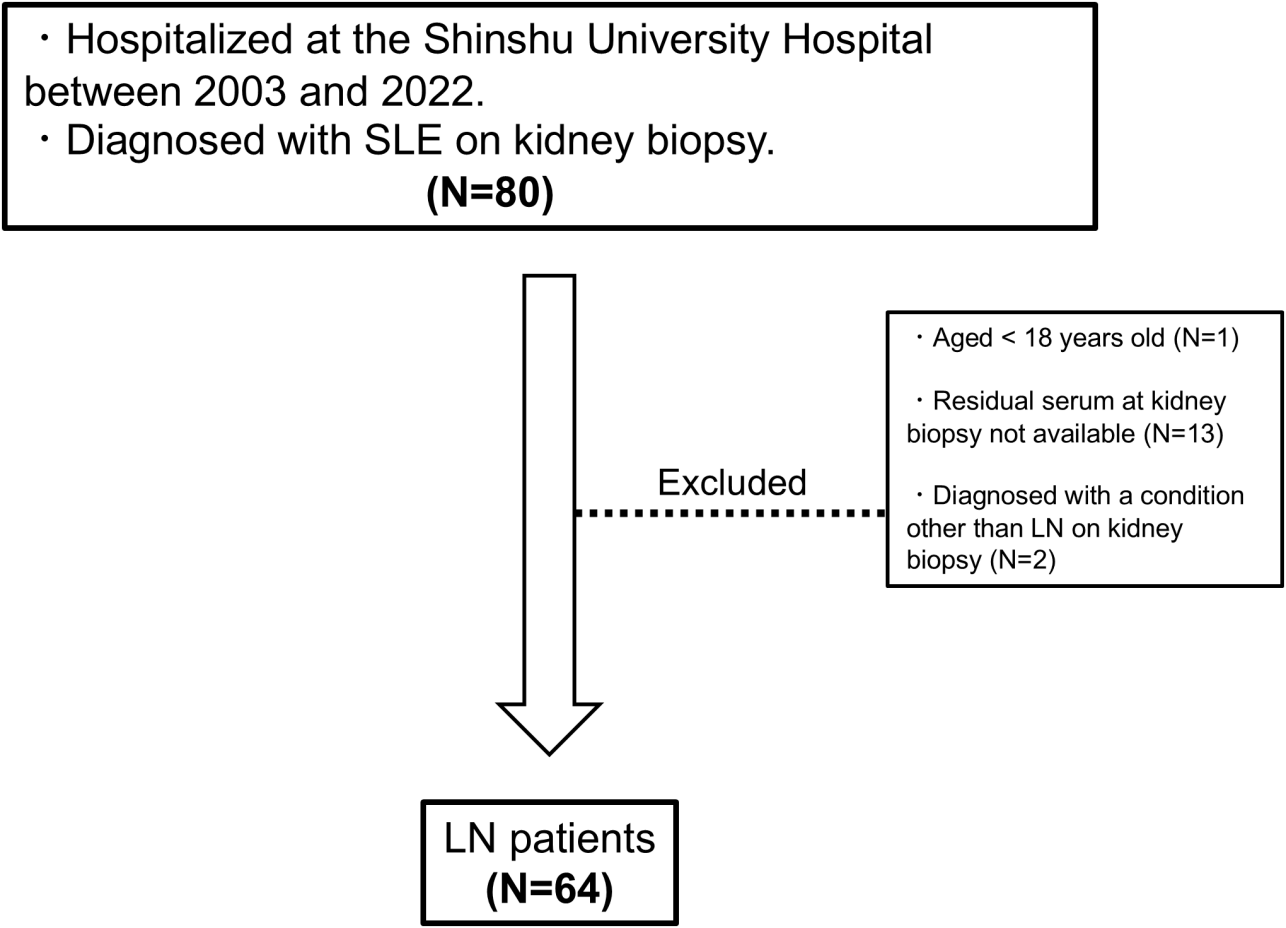
Study flowchart. LN, lupus nephritis; SLE, systemic lupus erythematosus.

**Figure 2:**
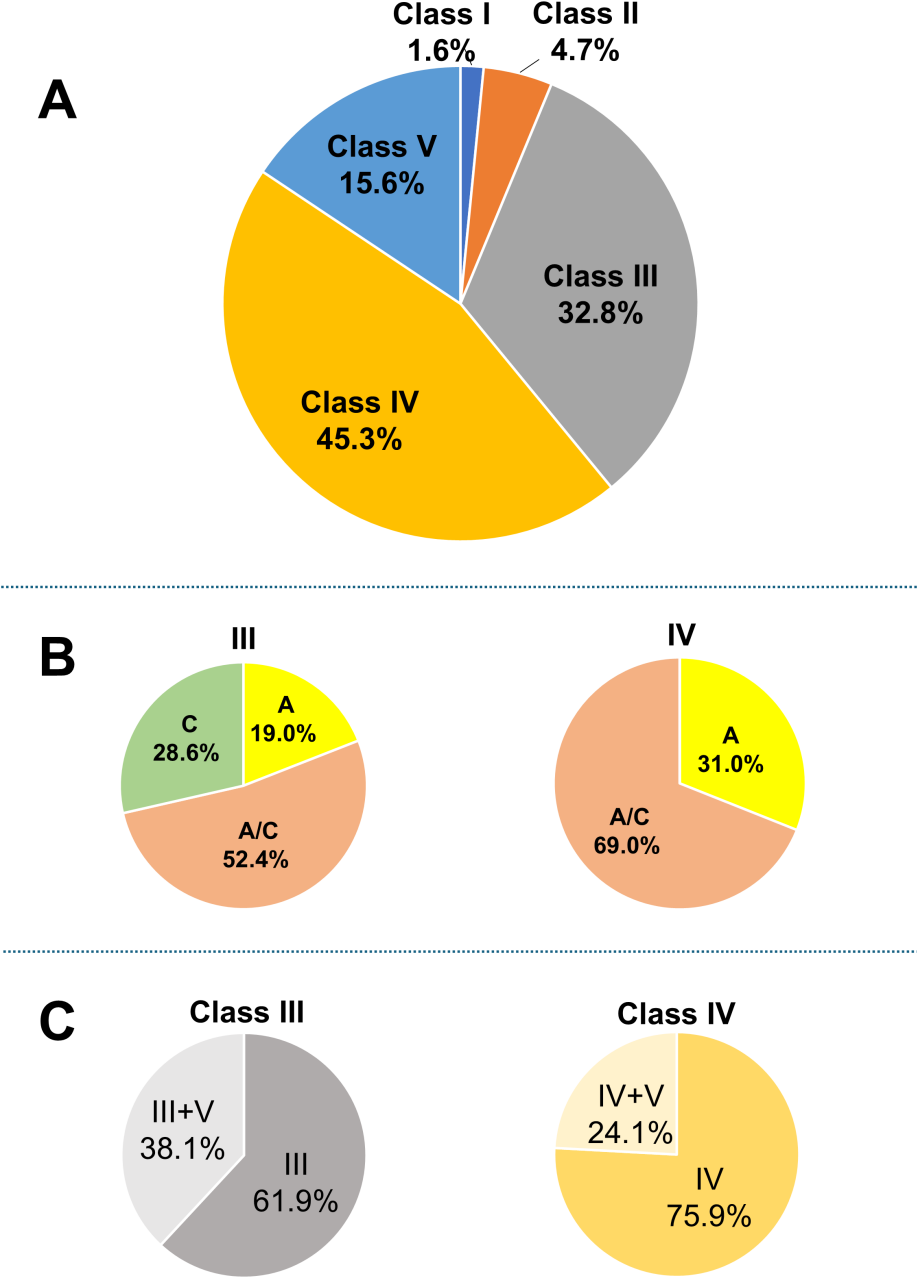
Breakdown of LN patients. **(A)** Breakdown of LN patients based on the ISN/RPS classification; **(B)** proportions of patients classified as A, A/C, and C; and **(C)** proportions of patients with overlapping Class V among those with Class III and IV LN. Based on the original ISN/RPS classification, patients with only active lesions are classified as A, those with only chronic lesions as C, and those with both lesion types as A/C. ISN/RPS, International Society of Nephrology/Renal Pathology Society; LN, lupus nephritis.

A flowchart of the study is provided in **Figure 1**. A total of 64 patients with LN were included in the analysis. The characteristics of the entire cohort of LN patients and the donor group (healthy controls) are summarized in **Supplementary Table 2**. All patients included in the study were ethnically Asian and held Japanese nationality; no individuals of other ethnicities, such as White, Black, or Hispanic, were enrolled. The mean age ± standard deviation (SD) of LN patients was 40.3 ± 14 years, with 12.5% male patients. The mean eGFR in LN patients was 74.2 ± 30 mL/min/1.73 m². Hematuria was observed in 64.6% of LN patients, and urine protein excretion was approximately 2 g/g Cr. **Table 1** and **Figure 2A** display the characteristics and distribution of LN patients categorized by the ISN/RPS classification, respectively. Class III and IV LN patients accounted for approximately 80% of the cohort, with no Class VI LN patients. The proportions of Class A, A/C, and C among Class III and IV LN patients are shown in **Figure 2B**, while the proportions of patients with overlapping Class V in Classes III and IV are presented in **Figure 2C**.

**Table 1:** Characteristics of patients with lupus nephritis categorized according to the International Society of Nephrology/Renal Pathology Society classification.

Variable	I (N=1)	II (N=3)	III (N=21)	IV (N=29)	V (N=10)	Donor (N=23)	*P-*value
Age (years)	34	51 ± 13	40 ± 14	39 ± 13	43 ± 18	57 ± 8	<0.001
Male (n)	0 (0.0)	1 (33.3)	3 (14.0)	1 (3.4)	3 (30.0)	10 (43.5)	0.005
Body Mass Index (kg/m^2^)	21.5	20.0 [18.9, 23.2]	21.2 [19.0, 22.3]	22.8 [21.4, 24.6]	22.4 [21.4, 24.6]	23.2 [21.2, 24.5]	0.20
Mean blood pressure (mmHg)	88.3	75.2 [72.3,78.1]	86.7 [82.0, 105.0]	99.3 [83.0, 105.0]	80.7 [75.1, 92.3]	88.3 [79.3, 93.3]	0.03
Hypertension (n)	0 (0)	0 (0)	5 (23.0)	9 (31.0)	3 (30.0)	4 (17.4)	0.81
Diabetes mellitus (n)	0 (0.0)	1 (33.3)	1 (4.8)	0 (0.0)	1 (10.0)	3 (13.0)	0.10
Symptoms							
Rash (n)	1 (100.0)	1 (33.3)	8 (38.0)	12 (41.0)	5 (50.0)	0 (0)	<0.001
Arthritis (n)	0 (0.0)	0 (0.0)	4 (20.0)	8 (28.0)	1 (10.0)	0 (0)	0.04
Serositis (n)	1 (100.0)	0 (0.0)	2 (10.0)	2 (7.0)	0 (0.0)	0 (0)	0.07
Pulmonary involvement (n)	0 (0)	1 (33.0)	2 (10.0)	3 (10.0)	1 (10.0)	0 (0)	0.58
SLEDAI	14	14 ± 1	18 ± 7	19 ± 7	16 ± 3	N/A	0.52
Laboratory data							
Albumin (g/dL)	2.7	2.6 [2.5, 2.7]	3.0 [2.7, 3.7]	2.9 [2.4, 3.3]	3.1 [2.6, 4.1]	4.4 [4.1, 4.6]	<0.001
Creatinine (mg/dL)	0.59	0.46 [0.46, 0.51]	0.71 [0.62, 0.94]	0.80 [0.68, 1.27]	0.67 [0.59, 0.91]	0.72 [0.64, 0.81]	0.04
eGFR (mL/min/1.73m^2^)	93.0	116.0 ± 24.0	77.9 ± 31.7	64.6 ± 28.3	79.2 ± 11.5	75.2 ± 11.5	0.02
CRP (mg/dL)	0.03	0.29 [0.27, 1.57]	0.09 [0.03, 0.73]	0.10 [0.05, 0.36]	0.07 [0.02, 0.34]	0.03 [0.01, 0.08]	0.02
TC (mg/dL)	284]	216 [205, 226]	183 [146, 218]	222 [168, 257]	218 [172, 274]	201 [191, 234]	0.46
LDL-C (mg/dL)	175	154 [136, 171]	111 [89, 144]	116 [84, 143]	127 [88, 163]	115 [107, 143]	0.53
TG (mg/dL)	441	98 [92, 103]	138 [106, 189]	178 [145, 314]	195 [80, 324]	132 [83, 184]	0.10
White blood cell (/μL)	6000	4100 [3300, 5200]	4530 [3500, 6900]	4700 [3800, 5800]	5100 [3800, 6100]	5520 [4415, 6115]	0.80
Hemoglobin (g/dL)	14.8	10.4 ± 0.7	11.8 ± 1.8	11.4 ± 1.9	12.2 ± 2.1	14.2 ± 1.0	<0.001
Platelet (×10^4^/μL)	29.8	21.9 ± 4.6	21.3 ± 7.4	18.9 ± 7.0	20.0 ± 5.0	24.6 ± 5.0	0.08
Immunological data							
Complement 3 (mg/dL)	32	53 [43, 57]	44 [30, 72]	38 [29, 54]	70 [61, 78]	N/A	0.06
Complement 4 (mg/dL)	2.7	10.5 [8.9, 10.6]	8.3 [3.8, 14.8]	4.5 [2.5, 10.3]	12.9 [5.5, 17.8]	N/A	0.09
Complement hemolytic 50% (U/mL)	11.5	32.9 [32.1, 33.8]	28.1 [11.1, 43.5]	13.4 [4.3, 20.7]	35.6 [31.2, 45.9]	N/A	0.04
ANA≧ 1:80 (n)	0 (0)	2 (100.0)	17 (85.0)	21 (100.0)	10 (100.0)	N/A	0.02
ds-DNA antibodies (IU/mL)	2.1	9.1 [8.1, 10.1]	44.9 [25.3, 93.8]	116.0 [31.6, 296.0]	29.9 [10.3, 60.0]	N/A	0.02
Urinalysis							
Hematuria (n)	0 (0)	0 (0)	12 (57.0)	21 (72.0)	7 (70.0)	0 (0)	<0.001
Urine protein (g/gCr)	0.23	2.87 [2.18, 3.79]	1.47 [0.96, 3.64]	2.11 [1.31, 4.91]	2.00 [0.80, 3.07]	0.00 [0.00, 0.00]	<0.001
Medications at admission							
Prednisolone (mg/day)	0	15.0 [7.5, 17.5]	6.0 [0.0, 15.0]	7.5 [0.0, 15.0]	7.5 [0.0, 11.9]	N/A	0.83
Calcineurin inhibitor (n)	0	1 (33.3)	7 (33.3)	5 (17.2)	1 (10.0)	N/A	0.49
Mycophenolate mofetil (n)	0	0 (0)	1 (4.8)	4 (13.8)	0 (0)	N/A	0.62
Hydroxychloroquine (n)	0	0 (0)	2(9.5)	1 (3.4)	0 (0)	N/A	0.76
Mizoribine (n)	0	0 (0)	2 (9.5)	2 (6.9)	1 (10.0)	N/A	0.90

Continuous variables are presented as mean ± standard deviation or median [interquartile range], and categorial variables are presented as n (%). The *P*-value calculated using one-way analysis of variance for differences among groups are presented. Statistical significance is set at *P-*value < 0.05. ANA, antinuclear antibody; CRP, C-reactive protein; eGFR, estimated glomerular filtration rate; HDL-C, high-density lipoprotein cholesterol; LDL-C, low-density lipoprotein cholesterol; N/A, not assessed; SLEDAI, Systemic Lupus Erythematosus Disease Activity Index; TC, total cholesterol; TG, triglyceride.

### 3.2 Association of the SS levels with LN patients, including subgroups classified by ISN/RPS

**Figure 3:**
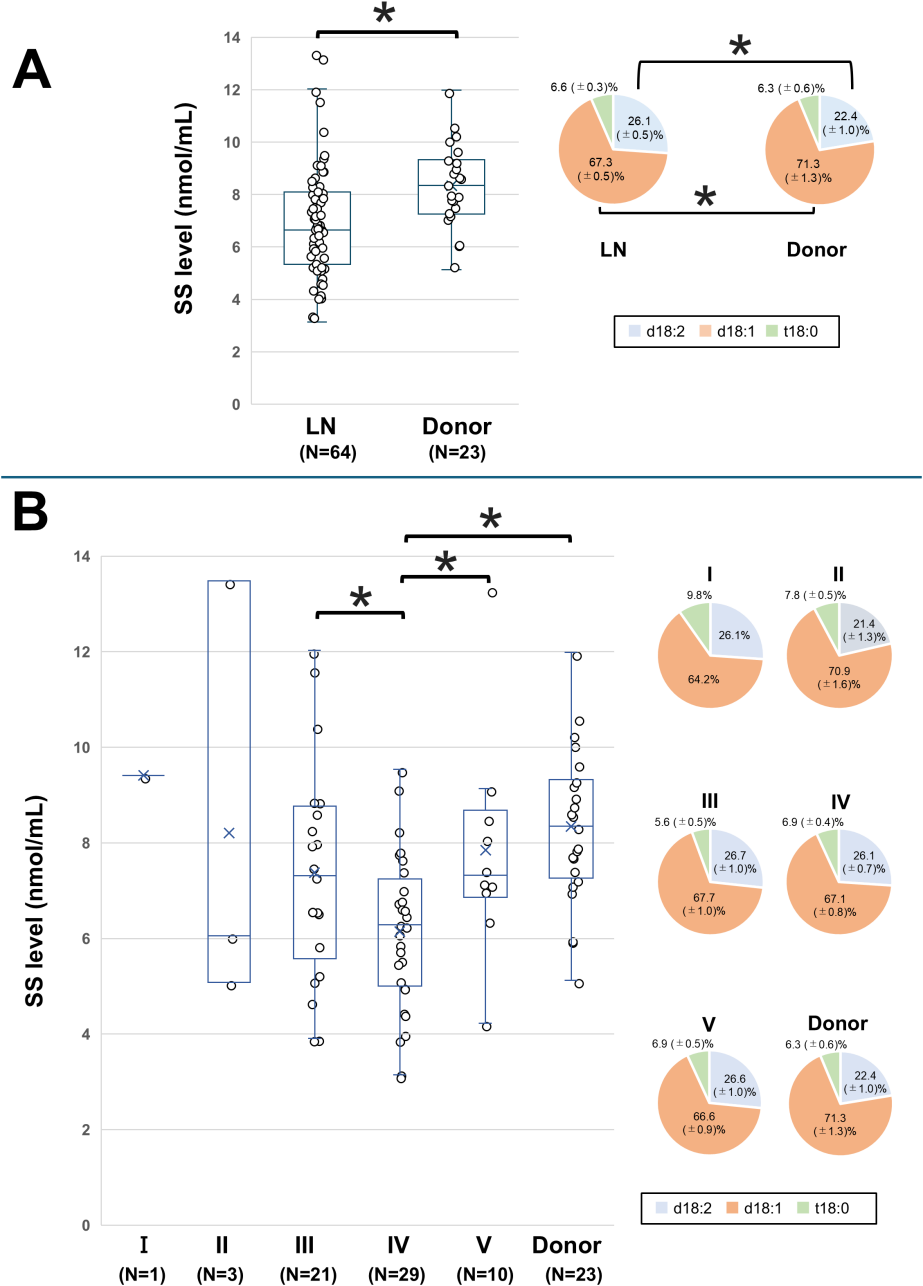
Comparison of SS levels between LN patients and the donor group. The SS level and proportions of SS species in **(A)** all LN patients and the donor group (healthy control) and in **(B)** LN patients classified according to the International Society of Nephrology/Renal Pathology Society classification and the donor group. The line within each box represents the median SS level. For comparisons of the SS level in **(B)**, overall group differences in SS levels were first assessed by one-way analysis of variance. Subsequently, pairwise comparisons between each group were performed using univariate and multivariate logistic regression models. Significant differences in SS levels between the disease groups are indicated by horizontal brackets with an asterisk. The SS species of each disease group are compared with those of the corresponding species in healthy controls. A *P*-value < 0.05 indicates statistical significance. d18:1, sphingosine; d18:2, sphingodienine; LN, lupus nephritis; SS, serum sulfatide; t18:0, phytosphingosine.

SS levels were compared between the donor group and all LN patients. The mean ± SD SS level in the donor group was 8.34 ± 1.68 nmol/mL, while in the LN group, it was 6.90 ± 2.22 nmol/mL, with the LN group exhibiting significantly lower levels (*P* = 0.007, **Figure 3A**). Sulfatide species analysis revealed lower d18:1 and higher d18:2 proportions in the LN group than in the donor group. No significant correlations were observed between SS levels and SLE activity parameters, including anti-dsDNA, complement levels, and SLEDAI (**Table 2**).

**Table 2:** Correlation between parameters related to SLE activity and SS levels.

Variable	*r*	*P-*value
ds-DNA antibodies (IU/mL)	−0.18	0.17
Complement 3 (mg/dL)	−0.01	0.96
Complement 4 (mg/dL)	0.19	0.14
Complement hemolytic 50% (U/mL)	0.11	0.41
SLEDAI	0.07	0.57

Pearson’s or Spearman’s rank correlation coefficient is used for correlation analysis of variables with normal or non-normal distribution, respectively. A *P*-value < 0.05 indicates statistical significance. SLEDAI, Systemic Lupus Erythematosus Disease Activity Index; SLE, systemic lupus erythematosus; SS, serum sulfatide.

The SS levels for Classes I–V, along with the donor group, are shown in **Figure 3B**. The mean ± SD SS level was 9.41 nmol/mL in Class I, 8.21 ± 1.68 nmol/mL in Class II, 7.33 ± 2.25 nmol/mL in Class III, 6.14 ± 1.63 nmol/mL in Class IV, 7.89 ± 2.12 nmol/mL in Class V, and 8.34 ± 1.68 nmol/mL in the donor group. The SS level in Class IV was significantly lower than those in Classes III and V and the donor group. These differences in SS levels between Class IV and Classes III, V, or the donor group remained significant even after adjusting for age and sex (**Supplementary Table 3**). The proportions of sulfatide species were similar across the LN classes, with no significant differences.

### 3.3 Association of the SS level with pathological active lesions in LN patients

**Figure 4:**
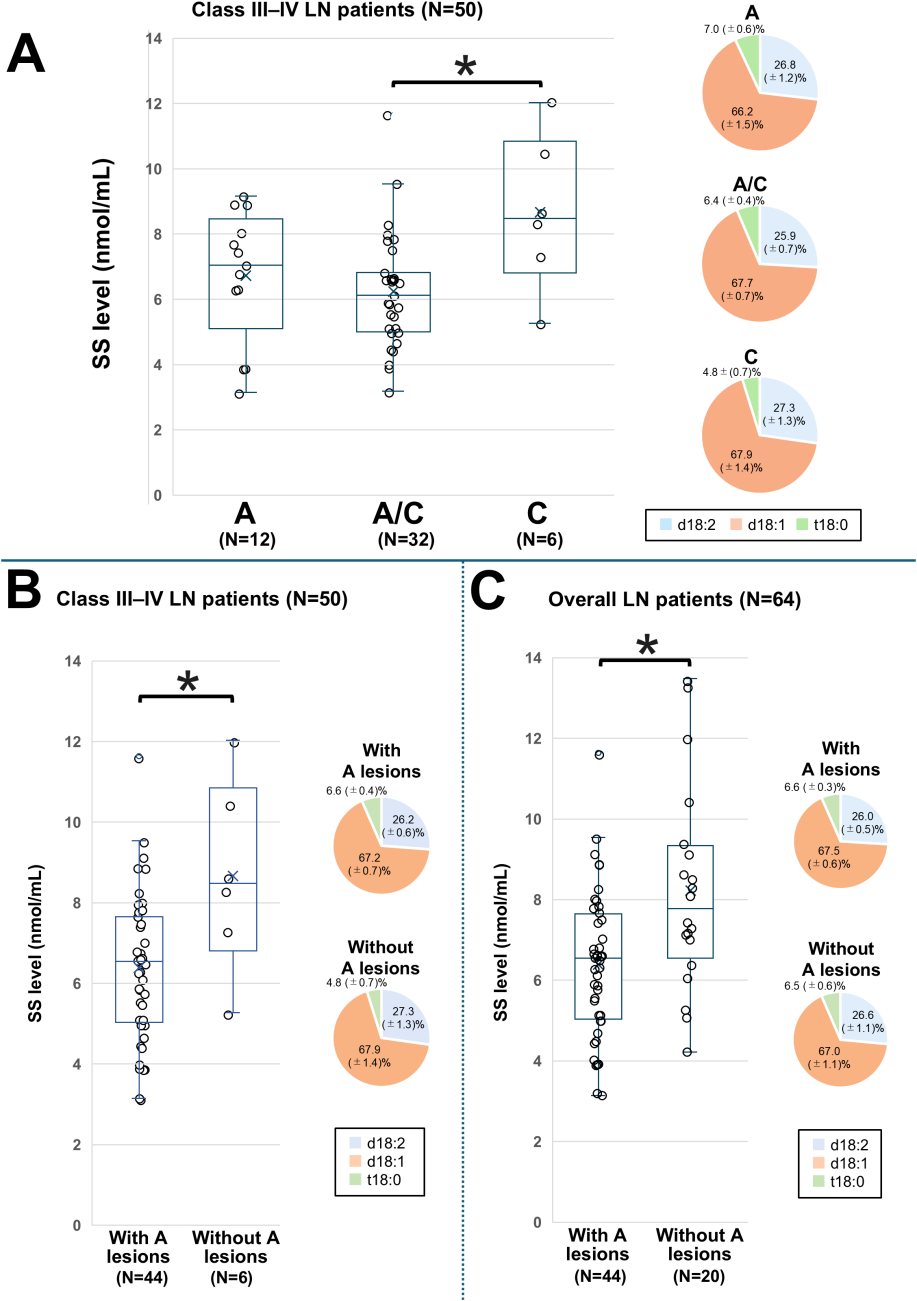
Comparison of the SS level based on the classification of active and chronic lesions in LN patients. SS levels and proportions of SS species among patients with LN stratified by pathological activity. Patients with only active lesions are classified into group A, those with only chronic lesions into group C, and those with both lesion types into group A/C. **(A)** Comparison among Class III–IV LN groups with A, A/C, and C; **(B)** Comparison between Class III and IV groups with active lesions and those without; and **(C)** Comparison between overall LN patients with active lesions and those without. The line within each box represents the median SS level. For comparisons in **(A)**, differences among the three groups were first assessed by one-way analysis of variance, followed by pairwise comparisons using univariate and multivariate logistic regression models. Significant differences in SS levels between the groups are indicated by horizontal brackets with an asterisk. A *P*-value < 0.05 indicates statistical significance. d18:1, sphingosine; d18:2, sphingodienine; LN, lupus nephritis; SS, serum sulfatide; t18:0, phytosphingosine.

The mean ± SD SS level was 6.73 ± 2.00 nmol/mL in group A, 6.24 ± 1.73 nmol/mL in group A/C, and 8.67 ± 2.37 nmol/mL in group C (**Figure 4A**). The SS level was significantly lower in group A/C compared to group C (*P* = 0.005), and this difference remained significant after adjusting for age and sex (**Supplementary Table 4**). We next divided patients with Class III–IV LN into two groups according to the presence (A + A/C) or absence (C only) of active lesions and conducted a comparative analysis. The corresponding patient backgrounds are summarized in **Supplementary Table 5**. Patients with active lesions showed high levels of ds-DNA, and low levels of serum complement, total cholesterol (TC), and low-density lipoprotein (LDL). **Figure 4B** shows the comparison of SS levels between these two groups. The presence of active lesions was significantly associated with lower SS levels (6.38 ± 1.81 vs 8.67 ± 2.37, *P* = 0.03). This association remained significant after adjusting for age, sex, eGFR, and albumin levels in Class III–IV patients (**Supplementary Table 6**).

Next, we assessed the association between SS levels and the presence of active lesions in all LN patients. The corresponding patient characteristics are presented in **Supplementary Table 7** and were similar to those of patients with Class III–VI LN. **Figure 4C** shows the comparison of SS levels between these two groups. SS levels were significantly lower in those with active lesions than in those without (6.38 ± 1.81 vs. 8.23 ± 2.55, *P* = 0.006), and this association remained significant after adjusting for the previously considered factors (**Supplementary Table 8**).

**Figure 5:**
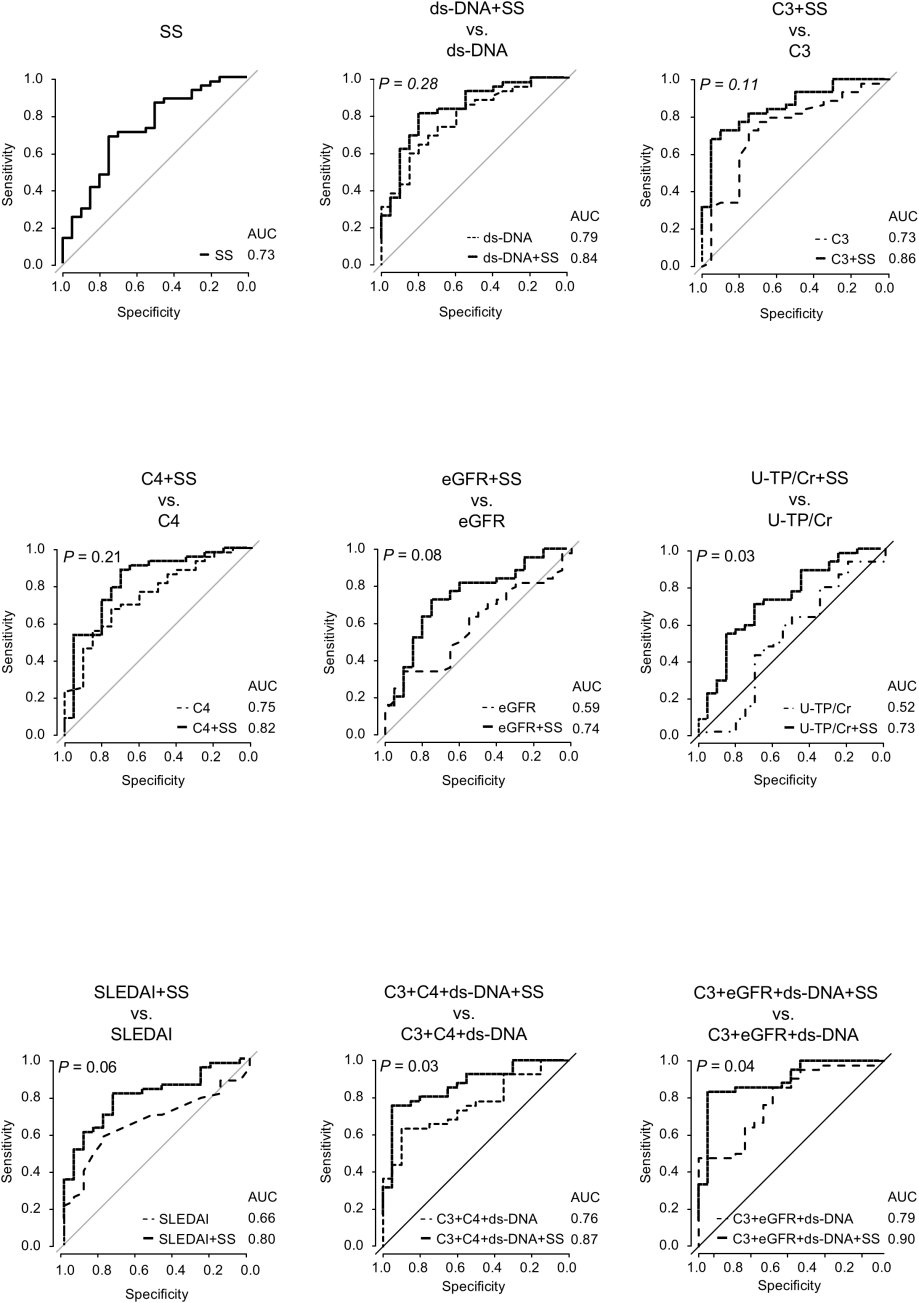
Receiver operating characteristic curve analyses for active lesion detection using SS level and other predictors in overall LN patients. Receiver operating characteristic curve analyses are conducted for active lesion detection using SS levels and other predictive markers in patients with LN. lines representing models that include SS are shown as solid black lines. *P*-values represent AUC comparisons between models with and without SS, using the DeLong’s test. A *P*-value < 0.05 indicates statistical significance. AUC, area under the curve; C3, complement 3; C4, complement 4; dsDNA, double-stranded DNA; eGFR, estimated glomerular filtration rate; LN, lupus nephritis; SS, serum sulfatide; U-TP, urinary total protein.

To evaluate the utility of SS levels in predicting active lesions, we performed ROC analyses of SS levels and other predictive markers in the overall LN population (**Figure 5**). Cutoff values, along with corresponding sensitivity and specificity for each predictor, are presented in **Supplementary Table 9**. The AUC for SS levels was 0.73 (95% confidence interval 0.59–0.86), which was similar to those of other predictors, but significantly higher than that of U-TP/Cr (*P* = 0.03). Combining SS levels with other predictors generally enhanced the predictive capability. The AUCs for combinations of SS with dsDNA, C3, C4, eGFR, and SLEDAI were higher than those for either assay alone. The AUC for the combination of SS and U-TP/Cr was similar to that of SS alone. Adding SS to formulas combining C3, C4, and dsDNA increased the AUC from 0.76 to 0.87. Similarly, adding SS to formulas with C3, eGFR, and dsDNA raised the AUC from 0.79 to 0.90, significantly enhancing detection ability.

**Figure 6:**
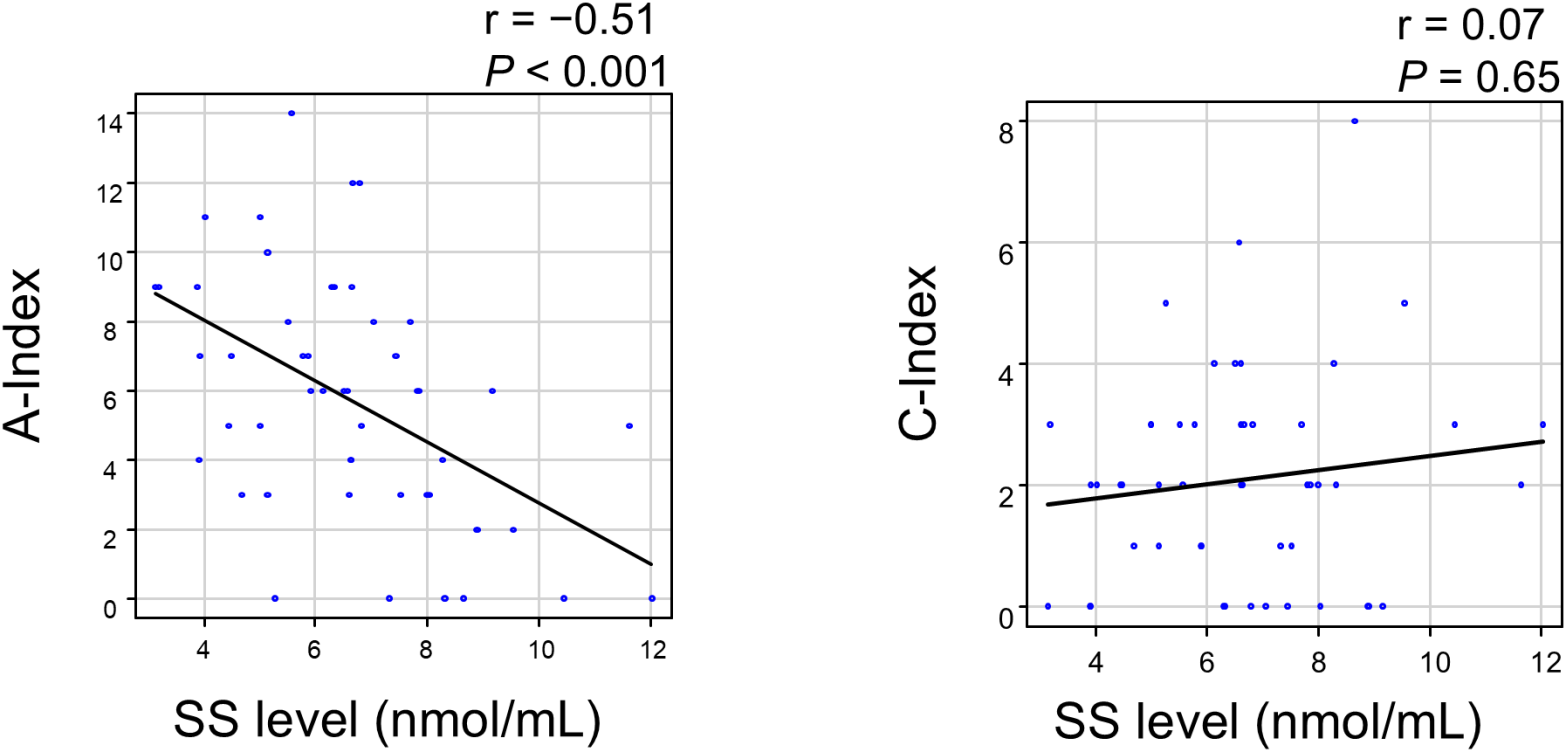
Correlation between the SS level and Activity/Chronicity Index in LN patients. A *P*-value < 0.05 indicates statistical significance. A-index, Activity Index; C-index, Chronicity Index; LN, lupus nephritis; SS, serum sulfatide.

**Figure 7:**
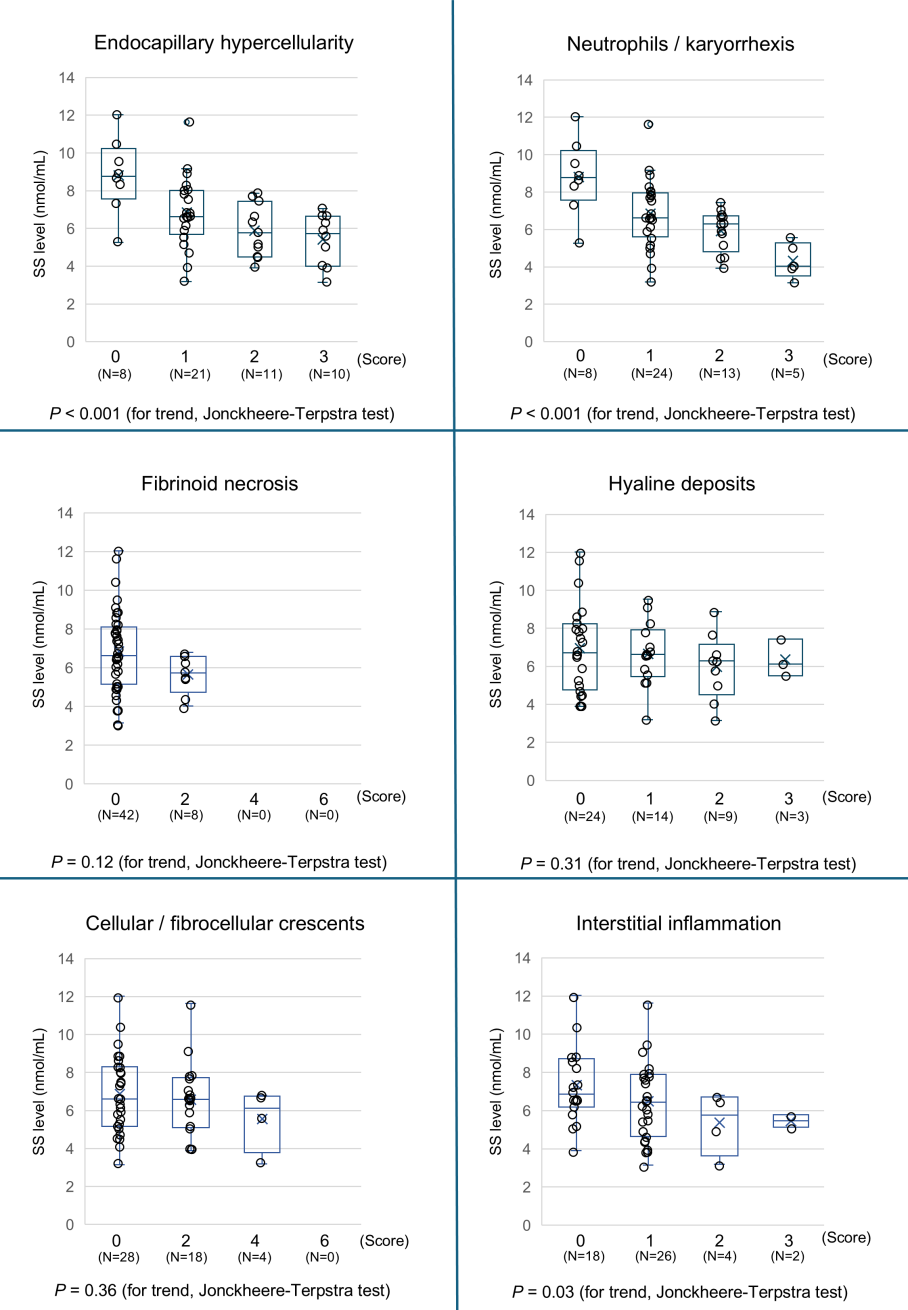
Association between the components of the Activity Index with the SS level. The SS levels in patients with LN are stratified by the Activity Index (0, 1, 2, 3 or 0, 2, 4, and 6) of each pathological score component, and the trends are assessed using the Jonckheere–Terpstra trend test. A *P*-value < 0.05 indicates statistical significance. LN, lupus nephritis; SS, serum sulfatide.

We then calculated the A-index and C-index and analyzed their correlation with SS levels. The A-index showed a strong negative correlation with SS levels, whereas the C-index showed no correlation (**Figure 6**). An analysis of the relationship between the A-index components and SS levels is presented in **Figure 7**. The Jonckheere–Terpstra test revealed significantly lower SS levels with higher scores for endocapillary hypercellularity, neutrophils/karyorrhexis, and interstitial inflammation.

### 3.4 Association of clinical outcomes and the SS level

**Table 3** shows the association between the clinical outcomes and SS levels. The follow-up rates were 82.8% at 1 year and 76.6% at 3 years, with no significant association found between prognostic outcomes and SS levels during either period.

**Table 3:** Association of clinical outcomes with SS levels at the 1-year and 3-year follow-ups.

1-year follow-up	Odds ratio	95% CI	*P*-value
Death	0.76	0.26–2.22	0.61
End-stage kidney disease	0.87	0.43–1.75	0.7
ΔeGFR < −30%	1.09	0.76–1.57	0.62
SLE flare	1.38	0.94–2.04	0.1
Thrombotic complications	1.1	0.63–1.93	0.74

3-year follow-up	Odds ratio	95% CI	*P*-value
Death	0.64	0.19–2.18	0.47
End-stage kidney disease	0.88	0.44–1.75	0.72
ΔeGFR < −30%	1.15	0.83–1.6	0.39
SLE flare	1.15	0.84–1.58	0.37
Thrombotic complications	0.9	0.53–1.54	0.71

The odds ratio of the SS level for each outcome is described with a 95% CI using univariate logistic regression analyses. The tracked patient percentages are 82.8% and 76.6% at the 1-year and 3-year follow-ups, respectively. CI, confidence interval; eGFR, estimated glomerular filtration rate; SLE, systemic lupus erythematosus; SS, serum sulfatide.

## 4 Discussion

In this study, SS levels were significantly lower in patients with LN than in healthy controls. Among the ISN/RPS classifications, the most pronounced decrease in SS levels was observed in Class IV LN. Additionally, SS levels were significantly lower in LN patients with pathologically active lesions, and a strong negative correlation was found between SS levels and the A-index. These findings suggest a strong association between low sulfatide levels and high disease activity in LN.

Previous studies have also reported a link between SS levels and various kidney vasculitis cases, including IgAV, AAV, and GBM, where SS levels were lower than those in healthy controls (12–14). While the pathophysiology of kidney vasculitis and LN differs, with kidney vasculitis characterized by autoantibodies (e.g., ANCA and anti-GBM) causing direct microvascular injury (19, 20), and LN involving immune complex deposition triggering complement activation and inflammation, both conditions lead to glomerular capillary inflammation (21–23). This suggests that similar underlying mechanisms contribute to the observed results in this study. While the precise mechanism between SS levels and LN remains unclear, several hypotheses have been proposed (12–14).

One hypothesis suggests that nephron loss or tubular injury, accompanied by elevated oxidative stress (OS), may suppress hepatic sulfatide synthesis in LN. Serum sulfatides are primarily synthesized in the liver, and previous animal studies have demonstrated that both the 5/6 nephrectomy model—characterized by marked nephron reduction—and the protein overload nephropathy model—characterized by tubular injury—are associated with increased systemic OS and a consequent decrease in hepatic sulfatide production (9, 24). Clinical studies also report a decrease in SS levels alongside increased OS in hemodialysis patients, which normalizes after kidney transplantation (25–27). Furthermore, OS is known to increase in SLE and LN (28), suggesting that nephron loss or tubular injury, and elevated OS in LN reduce SS levels by suppressing sulfatide synthesis in the liver. This mechanism may also partly explain the low serum lipid levels observed in patients with active lesions. Sulfatides, being a type of glycosphingolipid, have been reported to show a strong positive correlation with serum lipids (12, 14). Given their metabolic similarities, it is possible that they may share common regulatory pathways. Therefore, oxidative stress induced by highly active LN may suppress not only sulfatide synthesis but also the broader hepatic lipid synthesis, ultimately leading to the observed reduction in serum lipid levels. However, these hypotheses remain speculative at this point and require further validation.

An alternative hypothesis suggests that SS consumption occurs owing to its binding to overexpressed P-selectins on platelets during active vascular inflammation induced by LN. Sulfatides are abundant on platelet surfaces, where they regulate inflammation and thrombogenesis by promoting platelet adhesion and aggregation through their interaction with P-selectin (11, 29). During significant vascular inflammation, selectins such as P-selectin are overexpressed on both platelets and the vascular endothelium (30). Similarly, an increase in P-selectin expression has been observed in SLE and LN (31). The intravascular proliferation seen in LN may trigger microvascular inflammation, leading to the heightened expression of P-selectin. Consequently, the enhanced binding of SS to P-selectin could lead to its consumption, thereby reducing sulfatide levels in LN patients. In addition, since sulfatides are abundantly present in the kidney medulla (32) and exert anti-inflammatory effects through the modulation of natural killer T cells and myeloid dendritic cells (33, 34), decreased SS levels may reduce their renal availability, potentially exacerbating local inflammation and contributing to disease progression in LN.

In this study, Class IV LN patients exhibited the lowest SS levels compared with other class patients. Additionally, **Figure 3** shows a trend of lower SS levels predominantly in Class III–IV LN. This observation could be explained by the pathophysiology of the ISN/RPS classification and the patterns of immune complex deposition. Unlike other classes, Classes III and IV are marked by subendothelial deposits and capillary inflammation. As mentioned earlier, conditions characterized by significant capillary inflammation, such as kidney vasculitis, are associated with lower SS levels (12, 14). Thus, it is likely that SS levels are lower in Class IV, which is characterized by substantial intracapillary proliferation. These findings imply that measuring SS levels in LN patients could help estimate the presence of highly proliferative forms like Class IV LN. However, since this pilot study included a limited sample size, particularly for classes other than III–IV, the statistical power may have been insufficient. Further research is needed to explore the relationship between SS levels and other LN classes.

This study also revealed significantly lower SS levels in LN patients with active lesions, with a negative correlation between the A-index and SS levels. These findings suggest a robust association between SS levels and active lesions, which is consistent with findings in kidney vasculitis (12, 14). The established predictors of disease activity in SLE and LN include serum markers such as complement levels and ds-DNA antibodies, as well as clinical indices such as SLEDAI. Our findings suggest that SS levels operate independently of these predictors, implying that combining SS levels with these markers could improve the identification of active LN, as demonstrated by our results.

The identification of active LN is crucial. The 2021 KDIGO guidelines for the first time emphasized the need for clinicians to pay close attention to potentially reversible active lesions (35). If untreated, active LN can result in progressive renal impairment and poor short-term prognosis (36–38). Previous studies have shown that LN patients with an A-index > 2 face an increasing risk of poor renal outcomes and a higher likelihood of LN relapse after discontinuing maintenance immunosuppression (39–41). Conversely, active lesions in glomerular nephritis are often reversible with appropriate treatment (42, 43). This same association is observed in LN (44, 45), suggesting that SS measurement in LN patients is a valuable tool for identifying those who require aggressive treatment. However, this study does not directly address these important considerations, highlighting the need for further investigation.

In analyzing the trend of the A-index components and SS levels, a significant association was observed between endocapillary hypercellularity, neutrophils/karyorrhexis scores, and SS levels. These parameters reflect the degree of inflammatory cell proliferation within glomerular capillaries and the extent of the immune response, pointing to a strong relationship between the severity of intravascular inflammation and SS levels. Endocapillary inflammation could lead to interstitial injury either through direct extension of inflammation or indirectly owing to capillary ischemia, which may explain the observed connection between interstitial inflammation scores and SS levels. Although previous studies have reported a strong correlation between SS levels and crescent formation in kidney vasculitis, this was not observed in the present study (12, 14). One possible explanation is that only 44% of Class III–IV LN patients exhibited crescent formation, a notably lower proportion than that in other kidney vasculitis types. Furthermore, crescent formation was only focal in certain glomeruli in cases of LN. Likewise, for hyaline deposits and fibrinoid necrosis, most patients scored 0, suggesting insufficient statistical power. Nevertheless, further large-scale studies are needed to confirm these observations.

There are some limitations to this study. First, this was a single-center pilot study with a relatively small cohort of 64 patients. The limited sample size may have reduced the statistical power and generalizability of our findings. In particular, the number of patients with clinical outcome events during follow-up was small, and the representation of certain histological subtypes—such as Class I and Class II LN—was very limited (only one and three cases, respectively). This underrepresentation likely reflects current clinical biopsy practices, as Class I and II LN are typically associated with milder renal involvement and are therefore less likely to undergo kidney biopsy. As previously reported (45), more than 90% of biopsied LN cases are Class III–V, while Class I–II comprise only around 5% of all cases. Consequently, the small number of Class I and II cases limited our ability to assess SS levels across the full spectrum of LN severity. Furthermore, associations between SS levels and clinical endpoints (e.g., progression to ESKD, mortality, or relapse) could not be clearly established. However, we observed that lower SS levels were significantly associated with Class IV LN, which is widely known to be linked to poor kidney prognosis (46–48). This suggests that future studies with larger and more diverse cohorts may clarify the relationship between SS levels and long-term kidney outcomes. To overcome this selection bias and improve statistical robustness, future multi-center studies with broader LN class representation are warranted. Second, because this was a retrospective observational study, unknown or unmeasured confounding factors may have influenced the results. Despite our efforts to adjust for relevant clinical parameters, residual confounding cannot be excluded. Third, the study did not include a control group of SLE patients without LN. Since SLE can involve multiple organs, this study was unable to determine whether the fluctuations in SS levels were solely due to LN or influenced by other organ involvement. Given limitations in patient recruitment, we could not include SLE patients without LN, but this should be addressed in future studies. Fourth, we did not assess longitudinal changes in SS levels. Serum samples were collected only at the time of kidney biopsy, and follow-up samples were not available. As such, we were unable to evaluate whether SS levels dynamically reflect disease progression, remission, or response to treatment. Given the potential value of SS as a disease monitoring biomarker, future prospective studies with serial sample collection at multiple time points will be essential to clarify this aspect.

In conclusion, SS levels were significantly lower in LN patients than in healthy controls, with the lowest levels found in Class IV LN patients. SS levels were also strongly associated with active lesions and exhibited a notable negative correlation with the A-index. These findings suggest that SS levels serve as a valuable marker for estimating disease activity in LN. Further studies are necessary to better understand the clinical significance of SS levels in this context.

## Data Availability

The raw data supporting the conclusions of this article will be made available by the authors, without undue reservation.
