# Supplementary material for: Potential of serum sulfatide levels as a marker for classification and disease activity in lupus nephritis
Source: Front Immunol. 2025 Jun 16;16:1571147. doi: 10.3389/fimmu.2025.1571147 (PMC12206770; doi:10.3389/fimmu.2025.1571147)
Supplement: Supplementary file 1 [file DataSheet1.pdf]

# Supplementary Material

## 1 Supplementary Tables

### 1.1 Supplementary Table 1. Logistic regression formulas used for ROC analysis to predict active lesions.

| Model name | R formula used for logistic regression |
|---|---|
| SS | GLM.1 <- glm(Active.lesion ~ SS + Exam.date, family=binomial(logit), data=Dataset) |
| ds-DNA | GLM.2 <- glm(Active.lesion ~ ds.DNA, family=binomial(logit), data=Dataset) |
| ds-DNA+SS | GLM.3 <- glm(Active.lesion ~ ds.DNA + SS + Exam.date, family=binomial(logit), data=Dataset) |
| C3 | GLM.4 <- glm(Active.lesion ~ C3, family=binomial(logit), data=Dataset) |
| C3+SS | GLM.5 <- glm(Active.lesion ~ C3 + SS + Exam.date, family=binomial(logit), data=Dataset) |
| C4 | GLM.6 <- glm(Active.lesion ~ C4, family=binomial(logit), data=Dataset) |
| C4+SS | GLM.7 <- glm(Active.lesion ~ C4 + SS + Exam.date, family=binomial(logit), data=Dataset) |
| eGFR | GLM.8 <- glm(Active.lesion ~ eGFR, family=binomial(logit), data=Dataset) |
| eGFR+SS | GLM.9 <- glm(Active.lesion ~ eGFR + SS + Exam.date, family=binomial(logit), data=Dataset) |
| U-TP | GLM.10 <- glm(Active.lesion ~ UTP.Cr, family=binomial(logit), data=Dataset) |
| U-TP+SS | GLM.11 <- glm(Active.lesion ~ UTP.Cr + SS + Exam.date, family=binomial(logit), data=Dataset) |
| SLEDAI | GLM.12 <- glm(Active.lesion ~ SLEDAI, family=binomial(logit), data=Dataset) |
| SLEDAI+SS | GLM.13 <- glm(Active.lesion ~ SLEDAI + SS + Exam.date, family=binomial(logit), data=Dataset) |
| C3+C4+ds-DNA | GLM.14 <- glm(Active.lesion ~ C3 + C4 + ds.DNA, family=binomial(logit), data=Dataset) |
| C3+C4+ds-DNA+SS | GLM.15 <- glm(Active.lesion ~ C3 + C4 + ds.DNA + SS + Exam.date, family=binomial(logit), data=Dataset) |
| C3+eGFR+ds-DNA | GLM.16 <- glm(Active.lesion ~ C3 +eGFR +ds.DNA, family=binomial(logit), data=Dataset) |
| C3+eGFR+ds-DNA+SS | GLM.17 <- glm(Active.lesion ~ C3 +eGFR +ds.DNA + SS + Exam.date, family=binomial(logit), data=Dataset) |

C3, Complement 3; C4, Complement 4; ds-DNA, Double-stranded DNA antibodies; eGFR, estimated glomerular filtration rate; SLEDAI, Systemic Lupus Erythematosus Disease Activity Index; SS, Serum sulfatide; U-TP, Urinary total protein to creatinine ratio.

## 1.2 Supplementary Table 2. Patient characteristics

| Variable | LN (N=64) | Donor (N=23) | *P*-value |
|---|---|---|---|
| Age (years) | 40.3 ± 14.0 | 56.8 ± 8.4 | <0.001 |
| Male sex (n) | 8 (12.5) | 10 (43.5) | 0.005 |
| Body mass index (kg/m$^2$) | 21.9 [20.4, 24.0] | 23.2 [21.2, 24.5] | 0.32 |
| Mean blood pressure (mmHg) | 91.3 [80.0, 103.7] | 88.3 [79.3, 93.3] | 0.25 |
| Hypertension (n) | 17 (26.6) | 4 (17.4) | 0.57 |
| Diabetes mellitus (n) | 3 (4.7) | 3 (13.0) | 0.19 |
| Symptoms | | | |
| Rash (n) | 28 (43.8) | 0 (0) | <0.001 |
| Arthritis (n) | 14 (21.9) | 0 (0) | 0.02 |
| Serositis (n) | 5 (7.8) | 0 (0) | 0.32 |
| Pulmonary involvement (n) | 7 (10.9) | 0 (0) | 0.18 |
| SLEDAI | 17.7 ± 6.17 | N/A | — |
| Laboratory data | | | |
| Albumin (g/dL) | 3.0 [2.5, 3.4] | 4.4 [4.1, 4.6] | <0.001 |
| Creatinine (mg/dL) | 0.73 [0.60, 1.03] | 0.72 [0.64, 0.81] | 0.54 |
| eGFR (mL/min/1.73 m$^2$) | 74.2 ± 30.0 | 75.2 ± 11.5 | 0.87 |
| CRP (mg/dL) | 0.1 [0.03, 0.40] | 0.03 [0.01, 0.08] | 0.002 |

| | | | |
|---|---|---|---|
| TC (mg/dL) | 200 [166, 250] | 201 [191, 234] | 0.52 |
| LDL-C (mg/dL) | 118 [88, 150] | 115 [107, 143] | 0.78 |
| TG (mg/dL) | 155 [107, 316] | 132 [83, 184] | 0.06 |
| White blood cell (/μL) | 4805 [3718, 6143] | 5520 [4415, 6115] | 0.27 |
| Hemoglobin (g/dL) | 11.7 ± 1.9 | 14.2 ± 1.0 | <0.001 |
| Platelet ($\times 10^4$/μL) | 20.2 ± 7.7 | 24.6 ± 5.0 | 0.01 |
| Immunological data | | | |
| Complement 3 (mg/dL) | 44 [31, 69] | N/A | — |
| Complement 4 (mg/dL) | 5.9 [3.7, 13.2] | N/A | — |
| Complement hemolytic 50% (U/mL) | 19.3 [7.1, 35.7] | N/A | — |
| ANA ≥ 1:80 (n) | 50 (94.3) | N/A | — |
| ds-DNA antibodies (IU/mL) | 51.2 [12.7, 159.0] | N/A | — |
| Urinalysis | | | |
| Hematuria (n) | 41 (64.1) | 0 (0) | <0.001 |
| Urine protein (g/gCr) | 2.04 [1.13, 4.15] | 0.00 [0.00, 0.00] | <0.001 |
| Medications at admission | | | |
| Prednisolone (mg/day) | 6.75 [0.00, 15.00] | N/A | — |
| Calcineurin inhibitor (n) | 14 (21.9) | N/A | — |
| Mycophenolate mofetil (n) | 5 (7.8) | N/A | — |
| Hydroxychloroquine (n) | 3 (4.7) | N/A | — |
| Mizoribine (n) | 5 (7.8) | N/A | — |

Continuous variables are presented as mean ± standard deviation or median [interquartile range], and categorial variables are presented as n (%). Continuous variables are compared using either Student's t-test or the Mann–Whitney U test, depending on whether the variables are normally or non-normally distributed. Categorical variables are compared using Fisher's exact test. Statistical significance is set at *P*-value < 0.05. ANA, antinuclear antibody; CRP, C-reactive protein; eGFR, estimated glomerular filtration rate; HDL-C, high-density lipoprotein cholesterol; LDL-C, low-density lipoprotein cholesterol; N/A, not assessed; SLEDAI, Systemic Lupus Erythematosus Disease Activity Index; TC, total cholesterol; TG, triglyceride.

## 1.3 Supplementary Table 3. Regression analysis of SS level with 95% CI for pairwise disease group comparisons

| | Univariate | | Multivariate | |
|---|---|---|---|---|
| | Odds ratio (95% CI) | *P*-value | Odds ratio (95% CI) | *P*-value |
| II vs. III | 1.12 (−2.56, 4.82) | 0.53 | 0.63 (−3.68, 4.96) | 0.76 |
| II vs. IV | 2.05 (−0.55, 4.64) | 0.12 | 1.43 (−1.13, 4.01) | 0.26 |
| II vs. V | 0.61 (−4.41, 5.64) | 0.8 | 1.60 (−4.96, 8.17) | 0.59 |
| II vs. donor | 1.18 (−0.94, 3.31) | 0.26 | 1.39 (−0.60, 3.40) | 0.17 |
| III vs. IV | 1.27 (0.12, 2.42) | 0.03 | 1.37 (0.19, 2.55) | 0.02 |
| III vs. V | −0.47 (−2.30, 1.37) | 0.6 | −0.66 (−2.64, 1.30) | 0.5 |
| III vs. donor | −0.88 (−2.82, 1.05) | 0.36 | −0.86 (−2.79, 1.06) | 0.37 |
| IV vs. V | −1.75 (−3.13, −0.37) | 0.01 | −1.50 (−2.93, −0.06) | 0.04 |
| IV vs. donor | −2.64 (−4.46, −0.83) | 0.005 | −3.34 (−5.24, −1.42) | <0.001 |
| V vs. donor | −0.88 (−3.84, 2.08) | 0.55 | −0.39 (−3.66, 2.87) | 0.81 |

Both univariate and multivariate analyses (adjusted for age and sex) performed for regression analysis. CI, confidence interval; SS, serum sulfatide.

## 1.4 Supplementary Table 4. Regression analysis of SS level with 95% CI for pairwise comparison among three groups (A, A/C, and C)

| | Univariate | | Multivariate | |
|---|---|---|---|---|
| | Odds ratio (95% CI) | *P*-value | Odds ratio (95% CI) | *P*-value |
| A vs. A/C | 0.02 (−0.05, 0.10) | 0.56 | 0.02 (−0.05, 0.1) | 0.53 |
| A vs. C | −0.009 (−0.19, 0.01) | 0.08 | −0.11 (−0.22, 0.01) | 0.07 |
| A/C vs. C | −0.08 (−0.13, −0.02) | 0.006 | −0.08 (−0.13, −0.02) | 0.006 |

Both univariate and multivariate analyses (adjusted for age and sex) performed for regression analysis. CI, confidence interval; SS, serum sulfatide.

## 1.5 Supplementary Table 5. Patient characteristics by presence of active lesions in Class III–IV LN patients.

| Variable | With Active lesions (N=44) | Without Active lesions (N=6) | *P*-value |
|---|---|---|---|
| Age (years) | 39.1 ± 12.7 | 39.2 ± 17.9 | 0.99 |
| Male sex (n) | 3 (6.8) | 1 (16.7) | 0.41 |
| Body mass index (kg/$m^2$) | 21.6 [20.3, 23.7] | 22.2 [21.8, 25.0] | 0.33 |
| Mean blood pressure (mmHg) | 96.5 [81.6, 104.5] | 100.0 [88.7, 111.6] | 0.24 |
| Hypertension (n) | 11 (25.0) | 3 (50.0) | 0.33 |
| Diabetes mellitus (n) | 1 (2.3) | 0 (0.0) | 1.0 |
| Symptoms | | | |
| Rash (n) | 20 (45.5) | 1 (16.7) | 0.38 |
| Arthritis (n) | 13 (29.5) | 0 (0) | 0.32 |
| Serositis (n) | 4 (9.1) | 0 (0) | 1.0 |
| Pulmonary involvement (n) | 4 (9.1) | 1 (16.7) | 0.48 |

| | | | |
|---|---|---|---|
| SLEDAI | 18.7 ± 6.7 | 15.5 ± 6.3 | 0.27 |
| Laboratory data | | | |
| Albumin (g/dL) | 3.0 [2.6, 3.3] | 2.1 [2.0, 3.2] | 0.14 |
| Creatinine (mg/dL) | 0.74 [0.66, 1.1] | 0.95 [0.85, 1.04] | 0.27 |
| eGFR (mL/min/1.73 $m^2$) | 71.7 ± 31.3 | 59.3 ± 17.4 | 0.35 |
| CRP (mg/dL) | 0.1 [0.04, 0.41] | 0.06 [0.02, 0.26] | 0.41 |
| TC (mg/dL) | 183 [146, 240] | 333 [237, 386] | 0.004 |
| LDL-C (mg/dL) | 104 [82, 136] | 192 [141, 229] | 0.004 |
| TG (mg/dL) | 152 [107, 231] | 271 [161, 403] | 0.07 |
| White blood cell (/μL) | 4500 [3718, 5683] | 7190 [6913, 9970] | 0.02 |
| Hemoglobin (g/dL) | 11.5 ± 1.9 | 12.4 ± 1.5 | 0.24 |
| Platelet ($\times 10^4$/μL) | 19.0 ± 6.6 | 26.7 ± 8.6 | 0.01 |
| Immunological data | | | |
| Complement 3 (mg/dL) | 39 [29, 55] | 68 [58, 82] | 0.07 |
| Complement 4 (mg/dL) | 4.7 [2.8, 10.2] | 17.9 [10.2, 22.2] | 0.003 |
| Complement hemolytic 50% (U/mL) | 14.3 [4.5, 28.7] | 40.3 [36.1, 50.9] | 0.01 |
| ANA ≥ 1:80 (n) | 41 (93.2) | 5 (83.3) | 0.85 |
| ds-DNA antibodies (IU/mL) | 74.5 [28.4, 266.4] | 14.3 [6.0, 40.7] | 0.02 |
| Urinalysis | | | |
| Hematuria (n) | 30 (68.2) | 4 (66.7) | 1.0 |
| Urine protein (g/gCr) | 1.90 [1.15, 4.00] | 7.32 [1.84, 10.33] | 0.28 |

| | | | |
|---|---|---|---|
| Medications at admission | | | |
| Prednisolone (mg/day) | 1.00 [0.00, 10.00] | 8.00 [5.25, 13.75] | 0.3 |
| Calcineurin inhibitor (n) | 9 (20.4) | 3 (50.0) | 0.47 |
| Mycophenolate mofetil (n) | 4 (9.1) | 1 (16.7) | 0.68 |
| Hydroxychloroquine (n) | 2 (4.5) | 1 (16.7) | 0.38 |
| Mizoribine (n) | 3 (6.8) | 1 (16.7) | 0.58 |

Continuous variables are presented as mean ± standard deviation or median [interquartile range], and categorial variables are presented as n (%). Continuous variables are compared using either Student's t-test or the Mann–Whitney U test, depending on whether the variables are normally or non-normally distributed. Categorical variables are compared using Fisher's exact test. Statistical significance is set at *P*-value < 0.05. A, Active; ANA, antinuclear antibody; CRP, C-reactive protein; eGFR, estimated glomerular filtration rate; HDL-C, high-density lipoprotein cholesterol; LDL-C, low-density lipoprotein cholesterol; N/A, not assessed; SLEDAI, Systemic Lupus Erythematosus Disease Activity Index; TC, total cholesterol; TG, triglyceride.

## 1.6 Supplementary Table 6. Association of active lesions with the SS level in Class III–IV LN patients

| Model | Odds ratio (95% CI) | *P*-value |
|---|---|---|
| Crude model | 0.56 (0.35, 0.91) | 0.02 |
| Model 1 | 0.75 (0.31, 0.88) | 0.01 |
| Model 2 | 0.57 (0.35, 0.92) | 0.02 |
| Model 3 | 0.52 (0.30, 0.90) | 0.02 |
| Model 4 | 0.55 (0.31, 0.96) | 0.03 |

Odds ratios of the SS level for Class III–IV patients with active lesions adjusted for age and sex (Model 1); eGFR (Model 2); eGFR, age, and sex (Model 3); or eGFR, albumin, age, and sex (Model 4) and described with 95% CI values using univariate and multivariate logistic regression analyses. CI, confidence interval; eGFR, estimated glomerular filtration rate; LN, lupus nephritis; SS, serum sulfatide.

## 1.7 Supplementary Table 7. Patient characteristics by presence of active lesions in Overall LN patients.

| Variable | With Active lesions (N=44) | Without Active lesions (N=20) | *P*-value |
|---|---|---|---|
| Age (years) | 39.1 ± 12.7 | 42.9 ± 16.7 | 0.32 |
| Male sex (n) | 3 (6.8) | 5 (25.0) | 0.10 |
| Body mass index (kg/m$^2$) | 21.6 [20.3, 23.7] | 22.2 [21.2, 25.4] | 0.26 |
| Mean blood pressure (mmHg) | 96.5 [81.6, 104.5] | 85.7 [76.5, 94.7] | 0.08 |
| Hypertension (n) | 11 (25.0) | 6 (30.0) | 0.76 |
| Diabetes mellitus (n) | 1 (2.3) | 2 (10.0) | 0.23 |
| Symptoms | | | |
| Rash (n) | 20 (45.5) | 8 (40.0) | 0.79 |
| Arthritis (n) | 13 (29.5) | 1 (5.0) | 0.05 |
| Serositis (n) | 4 (9.1) | 1 (5.0) | 1.0 |
| Pulmonary involvement (n) | 4 (9.1) | 3 (15.0) | 0.67 |
| SLEDAI | 18.7 ± 6.7 | 15.4 ± 4.1 | 0.05 |
| Laboratory data | | | |
| Albumin (g/dL) | 3.0 [2.6, 3.3] | 2.7 [2.1, 3.62] | 0.35 |
| Creatinine (mg/dL) | 0.74 [0.66, 1.1] | 0.67 [0.57, 0.96] | 0.3 |
| eGFR (mL/min/1.73 m$^2$) | 71.7 ± 31.3 | 79.7 ± 26.9 | 0.33 |
| CRP (mg/dL) | 0.1 [0.04, 0.41] | 0.1 [0.03, 0.32] | 0.52 |
| TC (mg/dL) | 183 [146, 240] | 236 [202, 333] | 0.004 |

| | | | |
|---|---|---|---|
| LDL-C (mg/dL) | 104 [82, 136] | 158 [118, 216] | 0.004 |
| TG (mg/dL) | 152 [107, 231] | 180 [101, 361] | 0.35 |
| White blood cell (/μL) | 4500 [3718, 5683] | 5770 [3958, 7085] | 0.08 |
| Hemoglobin (g/dL) | 11.5 ± 1.9 | 12.1 ± 1.9 | 0.20 |
| Platelet ($\times 10^4/\mu L$) | 19.0 ± 6.6 | 22.8 ± 9.3 | 0.07 |
| Immunological data | | | |
| Complement 3 (mg/dL) | 39 [29, 55] | 64 [51, 76] | 0.003 |
| Complement 4 (mg/dL) | 4.7 [2.8, 10.2] | 11.5 [6.9, 19.2] | 0.002 |
| Complement hemolytic 50% (U/mL) | 14.3 [4.5, 28.7] | 35.8 [31.0, 45.6] | 0.001 |
| ANA ≥ 1:80 (n) | 41 (93.2) | 18 (90.0) | 0.89 |
| ds-DNA antibodies (IU/mL) | 74.5 [28.4, 266.4] | 13.2 [6.1, 42.2] | 0.001 |
| Urinalysis | | | |
| Hematuria (n) | 30 (68.2) | 11 (55.0) | 0.4 |
| Urine protein (g/gCr) | 1.90 [1.15, 4.00] | 2.52 [0.89, 4.96] | 0.79 |
| Medications at admission | | | |
| Prednisolone (mg/day) | 1.00 [0.00, 10.00] | 5.5 [0.00, 13.12] | 0.64 |
| Calcineurin inhibitor (n) | 9 (20.4) | 5 (25.0) | 0.87 |
| Mycophenolate mofetil (n) | 4 (9.1) | 1 (5.0) | 0.74 |
| Hydroxychloroquine (n) | 2 (4.5) | 1 (5.0) | 0.88 |
| Mizoribine (n) | 3 (6.8) | 2 (10.0) | 0.68 |

Continuous variables are presented as mean ± standard deviation or median [interquartile range], and categorial variables are presented as n (%). Continuous variables are compared using either Student's t-test or the Mann–Whitney U test, depending on whether the variables are normally or non-normally

distributed. Categorical variables are compared using Fisher's exact test. Statistical significance is set at *P*-value < 0.05. ANA, antinuclear antibody; CRP, C-reactive protein; eGFR, estimated glomerular filtration rate; HDL-C, high-density lipoprotein cholesterol; LDL-C, low-density lipoprotein cholesterol; N/A, not assessed; SLEDAI, Systemic Lupus Erythematosus Disease Activity Index; TC, total cholesterol; TG, triglyceride.

## 1.8 Supplementary Table 8. Association of active lesions with the SS level in overall LN patients

| Model | | *P*-value |
|---|---|---|
| Crude model | 0.66 (0.49, 0.89) | 0.006 |
| Model 1 | 0.65 (0.48, 0.89) | 0.007 |
| Model 2 | 0.64 (0.47, 0.88) | 0.006 |
| Model 3 | 0.61 (0.44, 0.86) | 0.004 |
| Model 4 | 0.61 (0.43, 0.88) | 0.007 |

Odds ratios of the SS levels for all LN patients with active lesions adjusted for age and sex (Model 1); eGFR (Model 2); eGFR, age, and sex (Model 3); or eGFR, albumin, age, and sex (Model 4) and described with 95% CI values using univariate and multivariate logistic regression analyses. CI, confidence interval; eGFR, estimated glomerular filtration rate; LN, lupus nephritis; SS, serum sulfatide.

## 1.9 Supplementary Table 9. Cutoff values, sensitivity, and specificity for individual predictors in ROC analysis

| | Cutoff value | Sensitivity | Specificity |
|---|---|---|---|
| SS (nmol/mL) | 6.83 | 0.68 | 0.75 |
| ds-DNA (IU/mL) | 67.9 | 0.6 | 0.85 |
| C3 (mg/dL) | 52 | 0.73 | 0.75 |
| C4 (mg/dL) | 6.9 | 0.67 | 0.75 |

| | | | |
|---|---|---|---|
| eGFR (mL/min/1.73 $m^2$) | 50 | 0.34 | 0.9 |
| U-TP (g/gCr) | 4.03 | 0.8 | 0.35 |
| SLEDAI | 17 | 0.58 | 0.79 |

The cut-off value, sensitivity, and specificity are determined using the Youden index. C3, complement 3; C4, complement 4; eGFR, estimated glomerular filtration rate; SLEDAI, Systemic Lupus Erythematosus Disease Activity Index; ROC, receiver operating characteristic; SS, serum sulfatide; U-TP, urinary total protein.
